# Analysis of 206 whole‐genome resequencing reveals selection signatures associated with breed‐specific traits in Hu sheep

**DOI:** 10.1111/eva.13697

**Published:** 2024-06-21

**Authors:** Fuping Zhao, Rui Xie, Lingzhao Fang, Ruidong Xiang, Zehu Yuan, Yang Liu, Lixian Wang

**Affiliations:** ^1^ State Key Laboratory of Animal Biotech Breeding Institute of Animal Science, Chinese Academy of Agricultural Sciences Beijing China; ^2^ Department of Animal Genetics, Breeding and Reproduction, National Experimental Teaching Demonstration Center of Animal Science, College of Animal Science and Technology Nanjing Agricultural University Nanjing China; ^3^ Center for Quantitative Genetics and Genomics Aarhus University Aarhus Denmark; ^4^ Faculty of Veterinary and Agricultural Science The University of Melbourne Parkville Victoria Australia; ^5^ Joint International Research Laboratory of Agriculture and Agri‐Product Safety of Ministry of Education Yangzhou University Yangzhou China

**Keywords:** high prolificacy, Hu sheep, selection signature, whole‐genome sequencing data

## Abstract

As an invaluable Chinese sheep germplasm resource, Hu sheep are renowned for their high fertility and beautiful wavy lambskins. Their distinctive characteristics have evolved over time through a combination of artificial and natural selection. Identifying selection signatures in Hu sheep can provide a straightforward insight into the mechanism of selection and further uncover the candidate genes associated with breed‐specific traits subject to selection. Here, we conducted whole‐genome resequencing on 206 Hu sheep individuals, each with an approximate 6‐fold depth of coverage. And then we employed three complementary approaches, including composite likelihood ratio, integrated haplotype homozygosity score and the detection of runs of homozygosity, to detect selection signatures. In total, 10 candidate genomic regions displaying selection signatures were simultaneously identified by multiple methods, spanning 88.54 Mb. After annotating, these genomic regions harbored collectively 92 unique genes. Interestingly, 32 candidate genes associated with reproduction were distributed in nine genomic regions detected. Out of them, two stood out as star candidates: *BMPR1B* and *GNRH2*, both of which have documented associations with fertility, and a HOXA gene cluster (*HOXA1*‐*5*, *HOXA9*, *HOXA10*, *HOXA11* and *HOXA13*) had also been linked to fertility. Additionally, we identified other genes that are related to hair follicle development (*LAMTOR3*, *EEF1A2*), ear size (*HOXA1*, *KCNQ2*), fat tail formation (*HOXA10*, *HOXA11*), growth and development (*FAF1*, *CCNDBP1*, *GJB2*, *GJA3*), fat deposition (*ACOXL*, *JAZF1*, *HOXA3*, *HOXA4*, *HOXA5*, *EBF4*), immune (*UBR1*, *FASTKD5*) and feed intake (*DAPP1*, *RNF17*, *NPBWR2*). Our results offer novel insights into the genetic mechanisms underlying the selection of breed‐specific traits in Hu sheep and provide a reference for sheep genetic improvement programs.

## 1 INTRODUCTION

Hu sheep is an invaluable Chinese indigenous sheep germplasm resource and well‐known for its hyper‐prolificacy and the beautiful wavy lambskins (CNCAGR, 2012). They originated from Mongolian sheep and were introduced from the pastoral region of North China to the Taihu Lake basin about 900 years ago (CNCAGR, 2012; Geng et al., 2002). After being introduced to South China, Hu sheep are raised indoors throughout the year and have adapted to the local hot and humid climate (Abied, Bagadi, et al., 2020). After long‐time artificial and natural selection, they have possessed their breed‐specific features, such as precocious puberty, perennial oestrus, and high fecundity (Feng et al., 2018). Their specific features are different from Mongolian sheep that exhibit seasonal estrous and singleton breeding. Now, Hu sheep have been utilized as the maternal line in sheep crossbreeding programs, and are almost distributed across China.

In the course of selection, the beneficial allelic frequencies can rapidly increase under positive selection pressure and be fixed within a population. The distinctive imprints were just sculpted on the genome, which are called selection signatures (Qanbari & Simianer, 2014). Scanning these selection signatures is crucially important in animal genetics. The genomic regions under selection often harbor QTLs or genes influencing some economically important traits (Ceballos et al., 2018; Rubin et al., 2010; Zhao et al., 2015). Coupling with the development of high‐throughput sequencing technology, the reduction of sequencing cost makes it possible to detect selection signatures on the whole genome. Thus, the selection signature analysis can reveal genomic regions of interest for selection and explore the potential genetic mechanisms of phenotypic polymorphisms and adaption.

The statistical methods for detecting the selection signatures within a population can be classified into three primary groups (Qanbari & Simianer, 2014; Saravanan et al., 2020). The first group is a statistic identifying the site frequency spectrum to determine whether allele frequencies are concordant with the hypothesis of genetic neutrality. Such as Tajima's D (Tajima, 1989), Fay and Wu's H statistic (Fay & Wu, 2000) and the composite likelihood ratio test (CLR) (Nielsen, 2005). The second group consists of powerful tests that analyzes the level of linkage disequilibrium (LD) associated with the target haplotypes and/or alleles of selection. Such as extended haplotype homozygosity (EHH), relatively extended haplotype homozygosity (REHH) (Sabeti et al., 2002) and integrated haplotype score (iHS) (Voight et al., 2006). The third group is established on assessment of DNA polymorphism levels, which includes runs of homozygosity (ROH) (McQuillan et al., 2008) and pooled heterozygosity (H_p_) (Rubin et al., 2010). These methods have been successfully implemented to detect positive selection signatures in domestic animals (Liu et al., 2021; Saravanan et al., 2020; Shi et al., 2020; Zhao et al., 2015, 2016).

In previous studies, Hu sheep together with other sheep breeds, was used in inter‐populations analysis to identify the genomic region showing evidence of positive selection not within Hu sheep itself (Abied, Bagadi, et al., 2020; Abied, Xu, et al., 2020; Liu et al., 2016; Wei et al., 2015). The main aim of this study is to detect selection signatures only in the Hu sheep breed. We used whole genome re‐sequencing data of 206 individuals and applied three complementary methods (CLR, iHS and ROH) to identify genomic regions putatively under selection. To reduce the false positive rate, genomic regions simultaneously identified by multiple methods were considered as being under selection. Then bioinformatics analysis was also performed to explain the biological function of the candidate genes residing in genomic regions. Our findings will help to better elucidate the genetic mechanism of breed‐specific traits of Hu sheep, and provide a reference for the genetic improvement of Hu sheep.

## 2 MATERIALS AND METHODS

### 2.1 Ethics statement

All animal experiments in this study were fully approved by the Animal Care and Use Committee of the Institute of Animals Science, Chinese Academy of Agricultural Sciences (IAS‐CAAS) with the following reference number: IASCAAS‐AE‐03, September 2014.

### 2.2 Animal sampling and whole‐genome resequencing

Two hundred and six Hu sheep were randomly selected from a Hu sheep breeding farm in the Gansu province of China. Ear tissue samples were obtained. The genomic DNA of these samples was extracted using a standard phenol‐chloroform protocol.

The quantity and quality of genomic DNA were examined using a NanoDrop‐2000 device (Thermo Fisher Scientific, Wilmington, DE, USA) and by 1% agarose gel electrophoresis. For each sample, at least 3 μg genomic DNA was used for sequencing library construction with an average insert size of 450 bp. After the examinations, we generated paired‐end libraries for each eligible sample using standard procedures. Next, sequencing was carried out using a 2 × 150 bp pair‐end configuration on an Illumina HiSeq X. The average read sequence coverage was 6× for all samples. The depth ensured the accuracy of variant calling and genotyping and met the requirements for next‐step analyses.

### 2.3 Variant discovery and genotyping

The raw reads were trimmed by Trimmomatic v0.39 (Bolger et al., 2014), and then BWA v0.7.17 software (Li & Durbin, 2009) was used to map high‐quality trimmed reads to the domestic sheep reference genome (*Ovis aries*, Oar_v4.0, https://www.ncbi.nlm.nih.gov/assembly/GCF_000298735.2/). Samtools v1.15 (Li et al., 2009) was utilized to covert alignment sam files into bam format, sort, index and generated an mpileup file containing genotype depth and allelic depth. Picard v2.0.1 (Li & Durbin, 2009) was used to remove MarkDuplicates and avoid any influence on variant detection. After that, GATK v.4.0.2.1 (DePristo et al., 2011) was performed with SNP calling. To ensure the high quality of the result, the software VCFtools v0.1.17 (Danecek et al., 2011) was used to further filter the output of GATK. Only the SNPs on the autosome were retained for further analyses and other SNPs that did not meet the following standards were excluded: (i) mean sequencing depth > 2X (over all samples); (ii) maximum missing rate <0.05; (iii) a minor allele frequency >0.05; (iv) *p*‐value of Hardy–Weinberg equilibrium >1 × 10^−6^; and (v) only keep two alleles. The reference genome link is Oar_v4.0.

### 2.4 Selection signature detection

In this study, we employed three complementary methods to identify the selection signatures in the Hu sheep population, which included CLR, iHS and ROH detection.

#### 2.4.1 Composite likelihood ratio (CLR) statistic

CLR test compares a neutral model for allele frequency spectrum with a selective sweep model since a recent selective sweep can cause allele frequency spectra to depart from the expectation under neutrality and reduced genetic diversity (Nielsen, 2005). In the neutral model, the probability of allele frequency spectrum is derived from the background pattern of variation in the genome. The CLR statistic does not only evaluate the skewness of the frequency spectrum across multiple loci but also incorporates information on the recombination rate to distinguish selection from other demographic events (Nielsen, 2005). In this study, the SweepFinder2 software (DeGiorgio et al., 2016) was used to calculate CLR value per locus. To increase statistical power and reduce sampling noise, the whole genome was divided into 500 kb windows with a 250 kb overlap and the maximum CLR value in each window was used as the test statistic. Windows at the top 1% of the empirical distribution were selected as candidate regions.

#### 2.4.2 Integrated haplotype score (iHS)

As a representative for haplotype‐based methods, the iHS score is developed based on extended haplotype homozygosity (EHH), and compares the extent of LD between haplotypes carrying the ancestral and derived alleles. The ancestral alleles of sheep SNPs were downloaded from https://www.sheephapmap.org. For the iHS test, we used rehh package v3.0 of R software (Gautier & Vitalis, 2012) to calculate *i*HS value. The unstandardized integrated haplotype score (*i*HS) is computed using the equation $\text{uniHS}=\ln\left(\frac{i{\mathrm{HH}}_{A}}{i{\mathrm{HH}}_{D}}\right)$, where *i*HH_A_ and *i*HH_D_ represented the integrated EHH value of the ancestor allele and derived allele, respectively. Then, the iHS standardization was performed by setting: $i\mathrm{HS}=\frac{\text{uniHS}-\text{Mean}\left(\text{uniHS}\right)}{\mathrm{SD}\left(\text{uniHS}\right)}$, where Mean (uniHS) and SD (uniHS) respectively represent the mean and standard deviation of uniHS restricted to those markers with a similar derived allele frequency as observed at the target marker. When the *i*HS value is a large positive value, it indicates that the haplotype may be the same as the ancestor, while when the iHS value is a large negative value, it indicates that there may be a new derived haplotype (Voight et al., 2006). In the same manner, the genome was also divided into 500 kb windows with a 250 kb overlap and the averaged |*i*HS| value in each window was used as its test statistic. In order to calculate the *p*‐value at the genomic level, *i*HS score value per window was further transformed following the equation $P_{i\mathrm{HS}}=-\log_{10}\left[1-2,|,\Phi \left(i\mathrm{HS}\right)-0.5|\right],$ where $\Phi \left(x\right)$ represents the Gaussian cumulative distribution function (under neutrality), and $P_{i\mathrm{HS}}$ is the two‐sided *p*‐value related to the neutral hypothesis. The $P_{i\mathrm{HS}}$ surpassing the top 1% of the empirical distribution were considered as candidate regions.

#### 2.4.3 ROH detection

In this study, we conducted ROH detection with consecutive runs module in detectRUNS 0.9.6 R package (Biscarini et al., 2018). The following criteria were selected for ROH detection: (i) the minimum length of one ROH was set 300 kb to avoid the impact of strong LD, (ii) no more than two missing SNP and one heterozygous genotype were allowed per ROH, (iii) the max gap between two consecutive ROH was set 500 kb, (iv) the minimum SNP number (*l*) required in one ROH was set to 88, which was computed using the equation proposed by Lencz et al. (2007), $l=\frac{\ln\left[\alpha /\left(n_{s}\times n_{i}\right)\right]}{\ln\left(1-\mathrm{het}\right)}$, where *α* is the false positive rate of ROH identified (set to 0.05 in the present study), $n_{s}$ is the number of SNPs per individual, $n_{i}$ is the number of individuals and *het* is the proportion of heterozygosity across all SNPs.

To identify the high‐frequency ROH regions in the Hu sheep genome, we calculated the percentage of the occurrence of SNPs in ROH by counting the times of an SNP detected in an ROH across all individuals. In this study, the threshold for identifying the genomic regions most commonly associated with ROH was the top 0.1% SNPs observed in ROH genomic regions.

### 2.5 Functional annotation of genomic regions under selection

We retained the candidate genomic regions simultaneously detected by at least two methods mentioned above. Gene annotation for regions under putative selection was performed based on the domestic sheep reference (Oar_v4.0). The search for positional candidate genes was extended 50 kb up‐ and downstream from genomic regions identified based on the result of LD analysis. The biological function of the positional genes annotated was carried out by survey of relevant literature.

### 2.6 Enrichment analysis

The annotated genes were further perform Gene Ontology (GO) and Kyoto Encyclopedia of Genes and Genomes (KEGG) pathway enrichment analysis using the DAVID online software (http://david.abcc.ncifcrf.gov/home.jsp).

## 3 RESULTS

### 3.1 Basic statistic of resequencing data of Hu sheep

In this study, we generated an average of 53,417,468 clean reads for 206 WGS data, ranging from 26,631,650 to 148,454,839. After quality control, we obtained a total of 1,604,526 SNPs.

### 3.2 Population analysis

**FIGURE 1:**
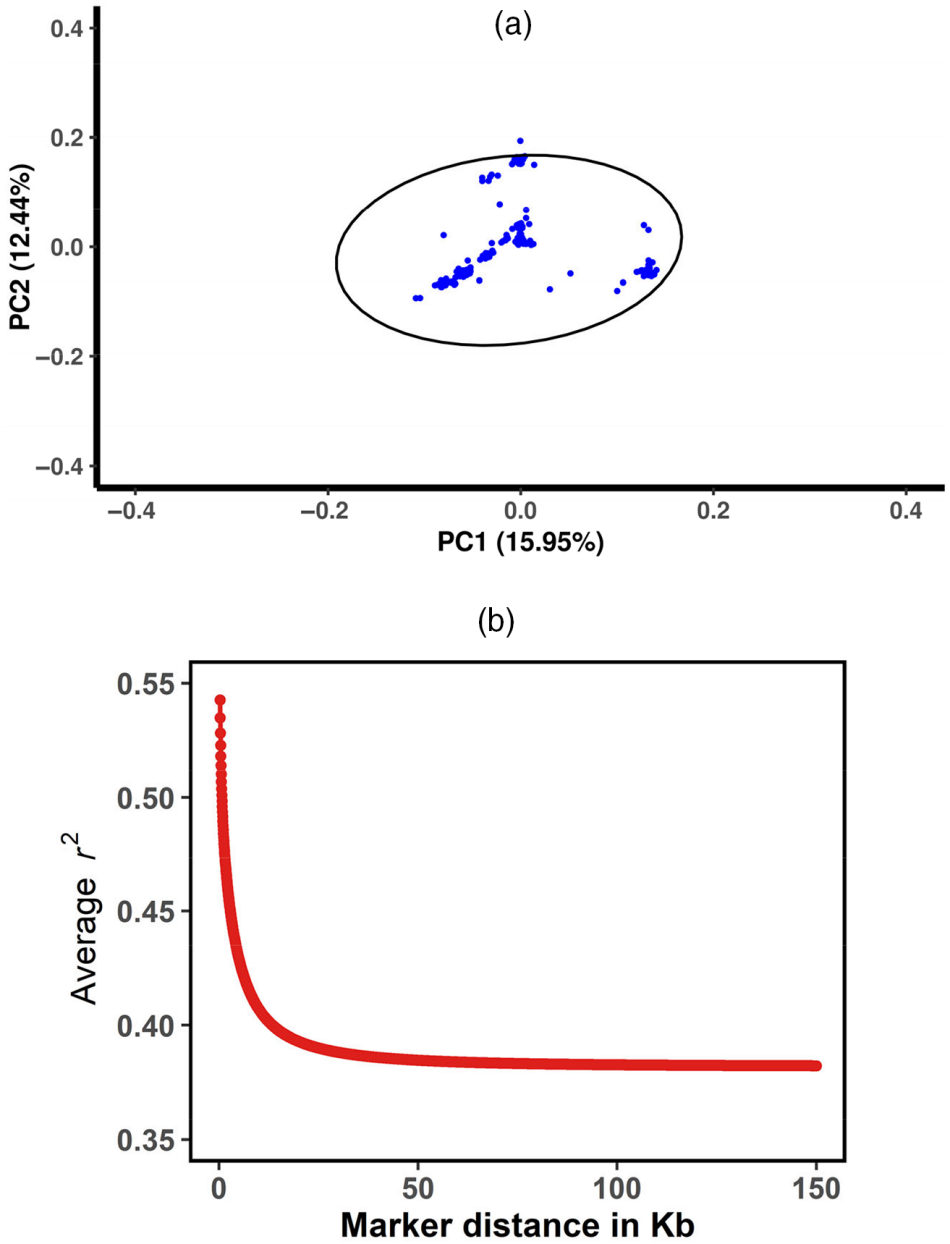
Population structure analysis. (a) Principal component analysis. (b) The decay of *r* ^2^ with pair‐wise SNP marker distances in Hu sheep.

PCA showed the total genetic variance in the population was explained by the first three principal components 15.95%, 12.44% and 9.78%, respectively. As seen in Figure 1a, individuals resided in the region of 99% confidence ellipse. Genomic relationships between all animals analyzed were calculated by Yang's method (Yang et al., 2011), and the heatmap was presented in Figure S1. Figure 1b displays the decay of average LD (*r^2^*) at various physical distances between two loci on all the autosomes in Hu sheep. The average *r* ^2^ at pair‐wise SNP distance of <5 Mb on autosomes ranged from 0.43 to 0.58. In addition, in this population, the expected and observed heterozygosity are 0.23 ± 0.11 and 0.27 ± 0.13, respectively. ROH‐based inbreeding values were 0.00898 ± 0.00823.

### 3.3 Genome‐wide scan of selection signature detection

**FIGURE 2:**
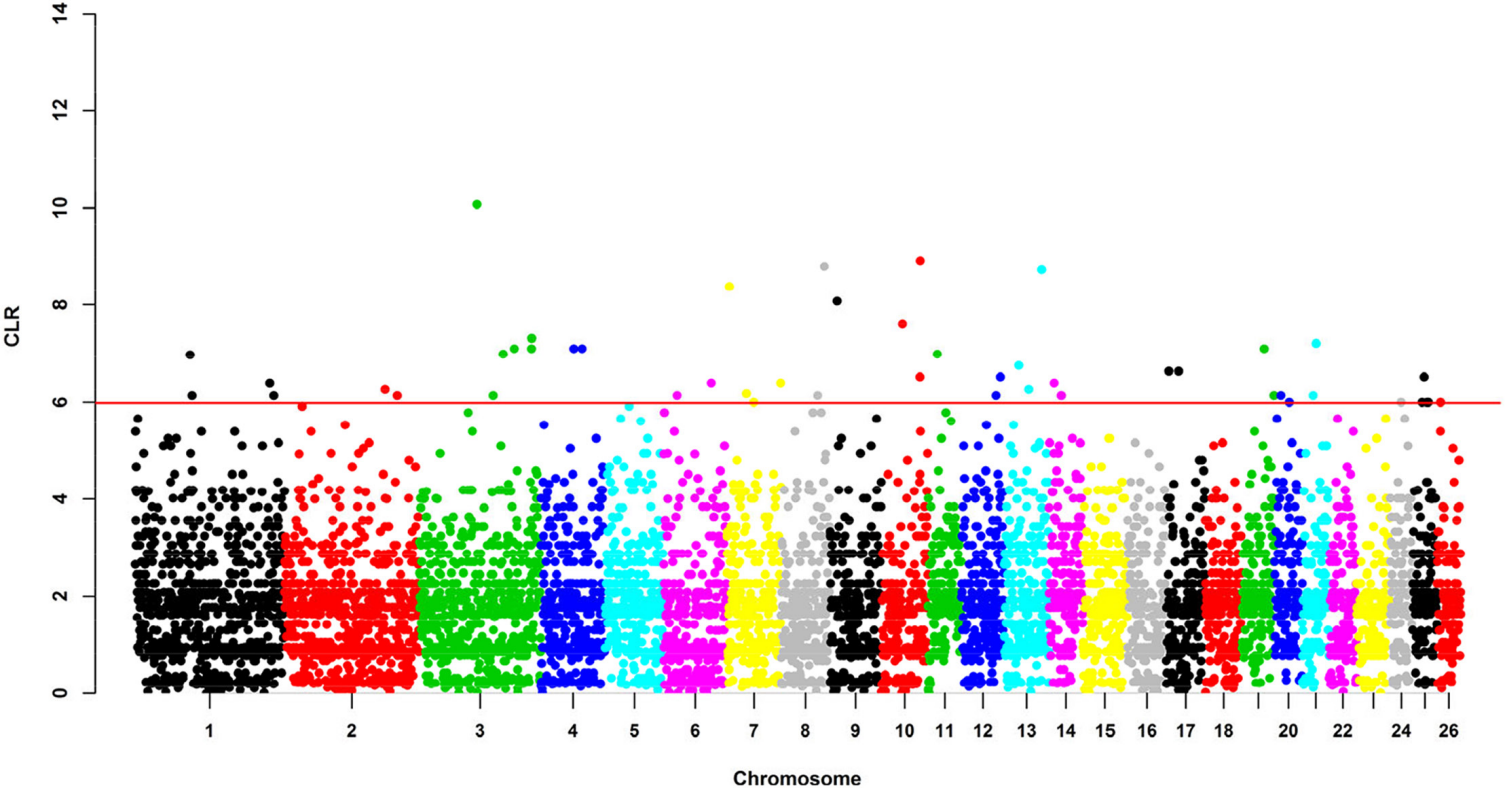
The distribution of selection signatures detected by CLR across the genome. The red line (5.98) was the threshold line of the candidate windows at the top 1% of the empirical distribution.

We employed three complementary methods to detect positive selection signatures within the Hu sheep population. The whole genome was split into a total of 9758 windows using the sliding window approach. Figure 2 visualized the genome‐wide distribution of the maximum CLR value per window. The average CLR value across the whole genome was 2.03, ranging from 0.00030 to 10.07. The highest CLR value (10.07) resided in OAR3: 105.5–106 Mb. Ninety‐seven windows surpassed the threshold value as candidate regions of positive selection among all windows in the Hu sheep genome (see Table S1).

**FIGURE 3:**
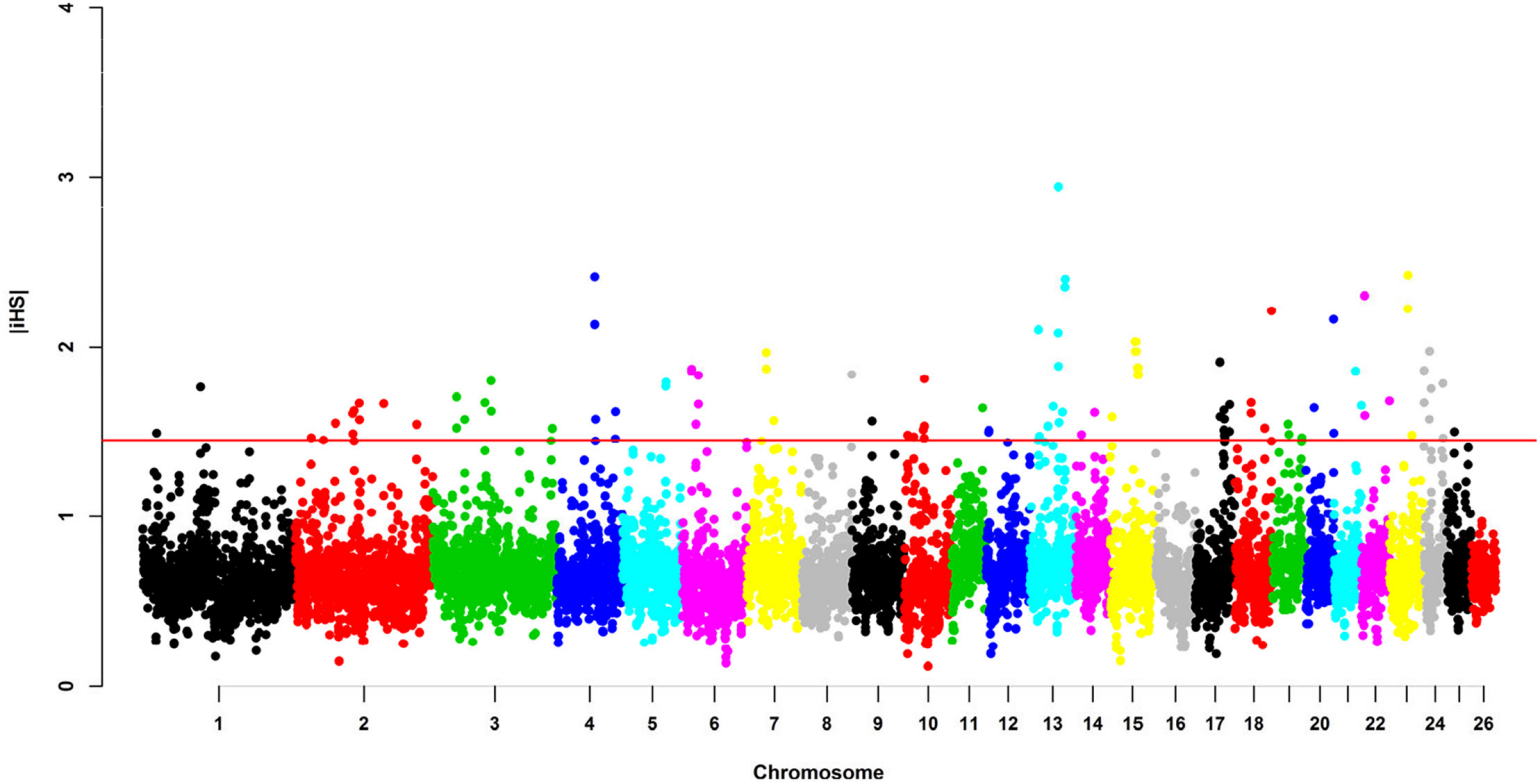
The distribution of selection signatures detected by iHS across the genome. The red line (1.45) was the threshold line of the candidate windows at the top 1% of the empirical distribution.

Figure 3 shows the distribution of |*i*HS| value per window along the Hu sheep genome. The mean |*i*HS| value of the whole genome was 0.68, with a range of 0.12 to 2.94. The highest value of mean |*i*HS| (2.94) occurred on OAR13: 51.25–51.75 Mb. Among all windows, 98 windows at the top 1% of the empirical distribution were selected as candidate regions (see Table S2).

Furthermore, we detected 9973 ROHs with a length of over 300 Kb among the 206 individuals. The number of detected ROHs varied among individuals (ranging from 2 to 356), with an average of 48.41 per individual. Among all these ROHs, the average length of ROH per individual was 22.00 Mb, accounting for 0.90% of the entire genome. The longest ROH was 3.25 Mb, which occurred on chromosome 21 and consisted of 362 SNPs, while the shortest ROH was 0.30 Mb, which occurred on chromosome 22 and consisted of 174 SNPs (see Table 1).

**TABLE 1:** Descriptive statistics for ROH in Hu sheep genome.

Sample size	SNP number	Average nength (Mb)		Average number	
		Mean ± SE	Range	Mean ± SE	Range
206	174–362	22.00 ± 1.40	1.05–191.5	48.41 ± 2.67	2–356

Abbreviations: SE, standard error; SNP Number, the range of SNP involved in ROH.

**FIGURE 4:**
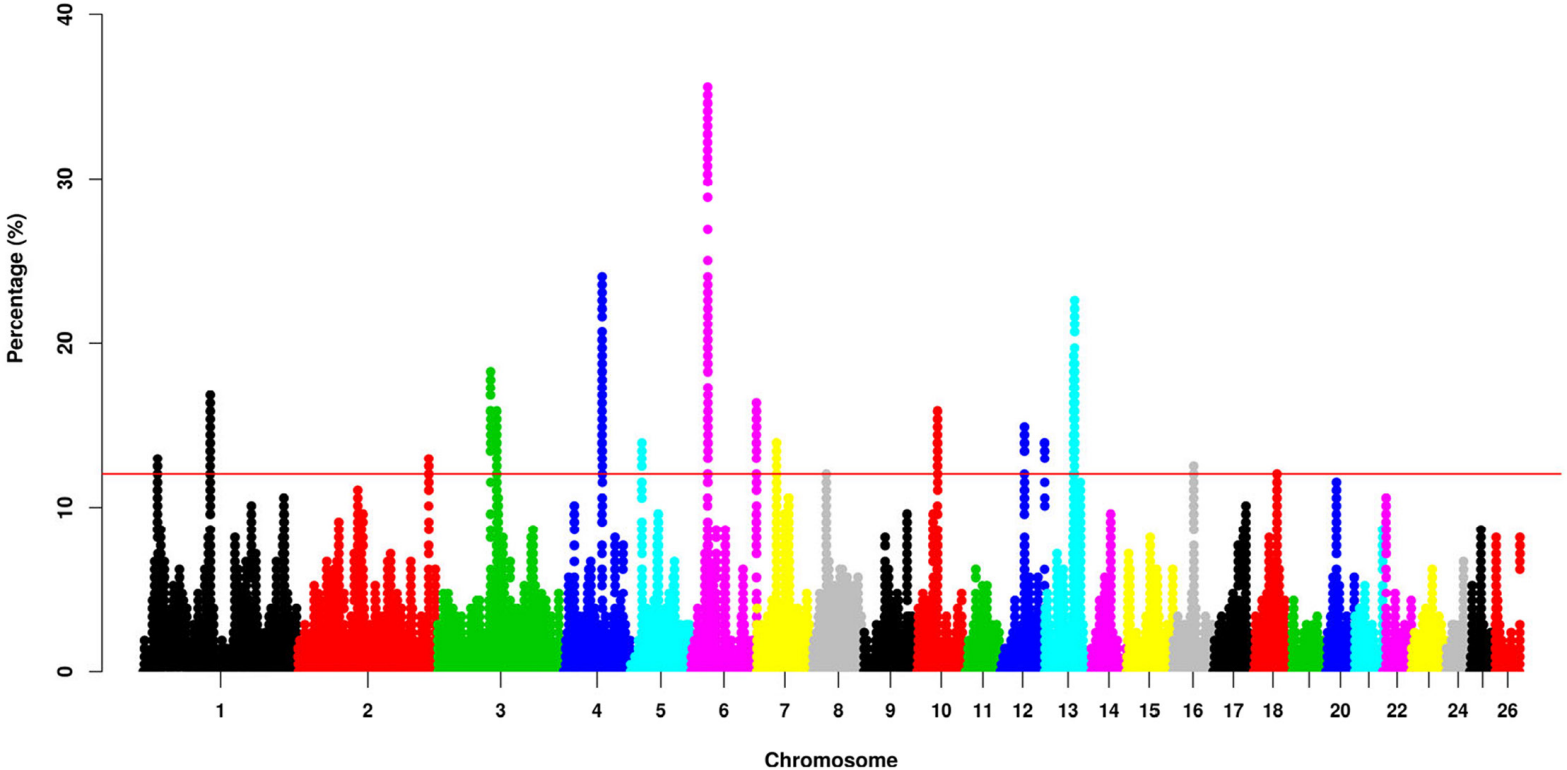
Manhattan plot of the occurrence (%) of SNPs in ROH across all individuals. The red line was the top 0.1% of SNPs observed in ROHs.

Figure 4 displayed the percentage of SNPs occurrence in ROHs along the genome of Hu sheep. As seen in Figure 4, ROH islands were mainly distributed on OARs 4, 6 and 13. ROH identified were unevenly distributed in the Hu sheep genome. Among all these ROHs, a total of 18 candidate genome regions were identified as high‐frequency ROH regions, representing 1497 SNPs with a range of 1 to 216 SNPs per region, and the total length of these regions was 5.17 Kb (see Table S3).

### 3.4 Identification and functional annotation of genes under positive selection

**FIGURE 5:**
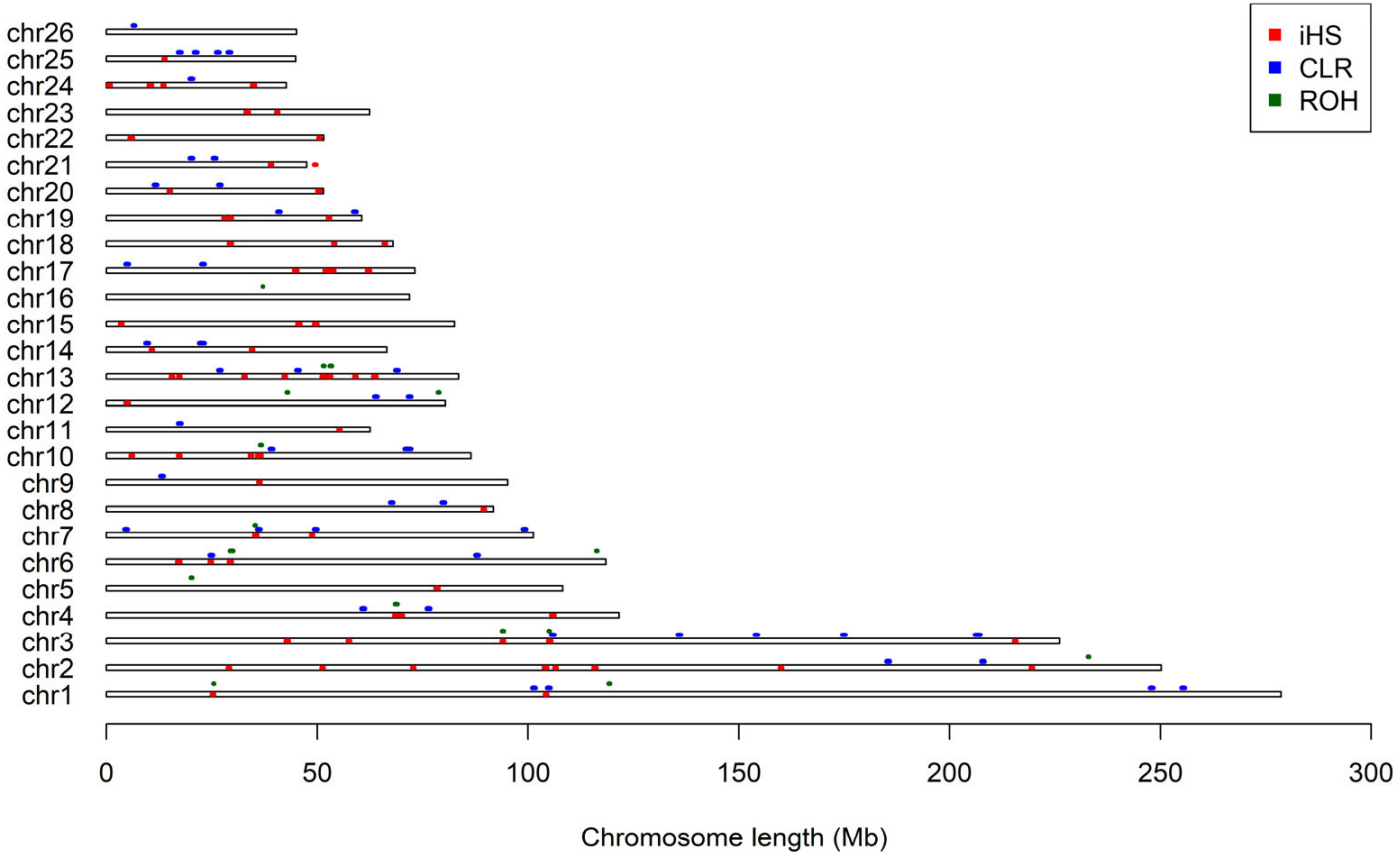
Distribution of genomic regions identified by three complementary test statistics across the Ovine chromosomes.

Figure 5 showed the distribution of genomic regions identified by three complementary statistics across the Ovine chromosomes. A total of 88.54 Mb genome regions were identified by at least one method and corresponding to 3.62% of the autosome in the Hu sheep genome. Among them, a total of 5.56 Mb genome regions were identified by both ROH and iHS tests; and a total of 0.5 Mb genome regions were identified by both iHS and CLR tests. However, there were no common genomic regions identified by both ROH and CLR tests (Figure 5).

Candidate regions for selection were defined as those genomic positions identified by multiple methods. Finally, 10 genome regions were retained for gene annotation, and the total length of these regions was 6.06 Mb (Table 2). After annotation, 93 unique genes were located in the selected genomic regions. We found 28 candidate genes (*CDKN2C*, *ACOXL*, *BCL2L11*, *HIBADH*, HoxA gene cluster (*HOXA1*‐*5*, *HOXA9*, *HOXA10*, *HOXA11* and *HOXA13*), *H2AFZZ*, *DNAJB14*, *UNC5C*, *BMPR1B*, *STARD9*, *PSPC1*, *RNF17*, *ATP12A*, *AVP*, *OXT*, *MRPS26*, *GNRH2*, *OPRL1*, *TCEA2*, *NPBWR2*, *SOX18*, *ABHD16B*, *ZGPAT* and *STMN3*) associated with fertility localized on nine genomic regions (OARs 1, 3, 4, 7 and 13) (Table 2). Among of them, two important star genes (*BMPR1B* and *GNRHER*) and the *HOXA* gene cluster were associated with reproductive resided in the OARs 4, 6 and 13, respectively.

**TABLE 2:** Genes located in genomic regions showing evidence of selection simultaneously identified by two methods in Hu sheep.

Chr	Start	End	Methods	Candidate genes
OAR1	25000001	25500001	iHS, ROH	*FAF1*, ***CDKN2C***, *TRNAY*‐*GUA*, *TTC39A*, *C1H1orf185*, *RNF11*
OAR3	93750001	94250001	iHS, ROH	*EXOC6B*
OAR3	104750001	105500001	iHS, ROH	***BCL2L11***, ***ACOXL***
OAR4	68250001	69002893	iHS, ROH	***HIBADH***, ***TAX1BP1*,** *JAZF1*, *EVX1*, ***HOXA1***, ***HOXA2***, ***HOXA3***, ***HOXA4***, ***HOXA5***, ***HOXA9***, ***HOXA10***, ***HOXA11***, ***HOXA13***
OAR6	24500001	25000001	iHS, CLR	*TRNAC*‐*GCA*, ***H2AFZ***, *LAMTOR3*, *DDIT4L*, *DAPP1*, ***DNAJB14***
OAR6	29250001	30022764	iHS, ROH	***UNC5C***, ***BMPR1B***, *PDLIM5*
OAR7	35000001	35500001	iHS, ROH	*CDAN1*, *CCNDBP1*, *EPB42*, ***STARD9***, *TTBK2*, *UBR1*, *TMEM62*
OAR10	36250001	36831220	iHS, ROH	*GJA3*, *TRNAG*‐*UCC*, *GJB2*, *ZMYM2*, ***PSPC1***, *PARP4*, *CENPJ*, *MPHOSPH8*, ***RNF17***, ***ATP12A***
OAR13	51250001	51750001	iHS, ROH	***AVP***, ***OXT***, ***MRPS26***, ***GNRH2***, *TMEM239*, *C13H20orf141*, *UBOX5*, *FASTKD5*, *VPS16*, *PCED1A*, *CPXM1*, *EBF4*, *PTPRA*
OAR13	52750001	53456146	iHS, ROH	*TRNAC*‐*GCA*, *GINS1*, *PCMTD2*, *MYT1*, ***OPRL1*, *TCEA2***, *UCKL1*, *DNAJC5*, *TPD52L2*, *ZBTB46*, *PRPF6*, ***NPBWR2***, *LKAAEAR1*, *RGS19*, ***SOX18***, *SAMD10*, *ZNF512B*, ***ABHD16B***, *LIME1*, *ARFRP1*, *TNFRSF6B*, *SRMS*, *PPDPF*, *EEF1A2*, ***ZGPAT***, *RTEL1*, ***STMN3***, *C13H20orf195*, *PTK6*, *KCNQ2*, *GMEB2*

*Note*: The genes in black and bald indicated associated with reproduction traits.

Moreover, we identified other genes that are related to hair follicle development (*LAMTOR3*, *EEF1A2*), ear size (*HOXA1*, *KCNQ2*), fat tail formation (*HOXA10*, *HOXA11*), growth and development (*FAF1*, *CCNDBP1*, *GJB2*, *GJA3*), fat deposition (*ACOXL*, *JAZF1*, *HOXA3*, *HOXA4*, *HOXA5*, *EBF4*), immune (*UBR1*, *FASTKD5*) and feed intake (*DAPP1*, *RNF17*, *NPBWR2*). Some of them were also enriched in the uterus development (see Table S4).

## 4 DISCUSSION

In this study, we employed three complementary statistics to identify the selection signatures within Hu sheep. These test statistical approaches are on the basis of different principles. The CLR test is based on hypothesis testing that compares the neutral model for the site frequency spectrum of a genomic window with the selective sweep model. This test had higher detection efficiency for fixed or to‐be‐fixed selection signals (Williamson et al., 2007), and is extremely sensitive to detecting selection signatures across multiple sites in a single population rather than analyzing each SNP separately. Compared to CLR, the iHS method is used to measure how the haplotypes are unusual around a core SNP relative to the whole genome. Hence, this method needs haplotype phasing, recombination map, genomic position, and ancestral and derived allelic information for each SNP. It should be mentioned that iHS method has a higher detection efficiency when the selected alleles are at moderate frequencies. Selecting for a beneficial mutation can emerge in a high‐frequency haplotype with extended linkage disequilibrium (LD). Along with the rapid increase in the frequency of a haplotype carrying a beneficial mutation, ROHs can emerge and lead to a regional reduction in genetic variation up‐ and downstream of the target locus. In a population, ROHs always enrich one genomic region carrying the target locus to form an ROH island. Therefore, the origin of ROH islands is also considered the result of recent positive selection (Ceballos et al., 2018; Pemberton et al., 2012). Hence, ROH can be also used to identify selection signatures, since the individuals that have been subjected to the selection pressure will exhibit long ROH around the target locus. Although few overlapping genomic regions were simultaneously identified by the three methods, we integrated these three different methods and found the overlapping genomic regions with selection signatures to increase the power of detection.

The length of ROHs can reflect the inbreeding history for one population, because the possibility of ROH being interrupted due to recombination is increased with the increase in the number of generation. The shorter ROHs are generally produced by more ancient inbreeding, while the longer ROHs indicate more recent inbreeding (Ceballos et al., 2018). In this study, long ROHs were not obtained using the whole‐genome sequencing data. The whole‐genome sequencing data can obtain all SNPs on the whole genome compared to the SNP chip. It was reported that high‐density chip data can detect shorter ROH fragments than low‐density chip data, while low‐density chips cannot detect most ROH fragments of 500 kb ~ 1 Mb (Purfield et al., 2012). In the present study, the minimum length of ROH was set to 300 Kb to avoid the impact of strong LD between genes. The lengths of ROHs centralized on the 300 Kb to 1 Mb, which was shorter than using SNP chip data. These short ROHs may indicate that the inbreeding events of Hu sheep in recent generations have not happened, or this pattern would be an effect of admixture or crossbreeding, which may be contributed to the breakdown of long ROH segments on domestic lines.

As a valuable sheep germplasm resource, Hu sheep is a typical Chinese indigenous hyper‐prolificacy sheep breed. Now, they are distributed across the whole country and are used as maternal lines in sheep crossbreeding programs. In this study, we identified two star genes and one gene cluster related to reproduction traits in sheep. One star genes is *BMPR1B* gene which is a paramount regulator of ovulation and litter size in sheep. *BMPR1B* had been documented to be involved in increasing the ovulation rate of ewes, and played an important role in follicle development (Liu et al., 2014). Moreover, this gene was previously detected associated to reproduction traits in the Hu sheep breed (Abied, Xu, et al., 2020; Liu et al., 2016; Wei et al., 2015; Zhang et al., 2017). The other star gene is *GNRH2* cirical for reproduction. *GNRH2* gene encoded the gonadotropin‐releasing hormone (GNRH) which triggered the sythesis and secretion of gonadotropins and played an important role in regulating the entire hypothalamic–pituitary‐gonadal axis (Desaulniers et al., 2017; Mijiddorj et al., 2017). MacColl et al. (2002) found that if the *GNRH* gene was mutated or its expression was suppressed, the secretion of various hormones in the hypothalamus‐pituitary‐gonadal axis would be blocked, which could lead to infertility. One gene cluster also identified on OAR4: 68.25–69.00 Mb is the HoxA gene cluster, which included 9 HoxA genes (*HOXA1*‐*5*, *HOXA9*, *HOXA10*, *HOXA11* and *HOXA13*). The HOXA gene cluster is an essential contributor to morphological differentiation of the female reproductive tract, cyclic endometrial changes, and embryo implantation (Du & Taylor, 2015). *HOXA3* gene was reported to be expressed in the bovine oocytes and early‐stage embryos and may influence oocyte maturation and the first stages of embryonic development (Paul et al., 2011). This gene also resided in ROH island in Chinese indigenous sheep breeds (Liu et al., 2021). *HOXA9*, *HOXA10*, *HOXA11*, and *HOXA13* are responsible for modulating female reproductive organs in mammalian species. *HOXA9* modulates the development of the oviduct, both *HOXA10* and *HOXA11* are jointly culpable for the uterus formation, while *HOXA11* and *HOXA13* are responsible for the development of the anterior vagina and cervix (Ekanayake et al., 2022).

Other candidate genes related to reproduction were found as well. *AVP* may associate with the abnormal secretion of GNRH (Tan et al., 2021). *OXT* was related to litter size in Yunnan semi‐fine wool sheep (Guo et al., 2022). *ZGPAT* may influence protein synthesis, processing and secretion so as to regulate milk production in yak (Xin et al., 2020). *ACOXL* gene was reported that played a pivotal role in fertility and precocity (Sbardella et al., 2021). Some candidate genes are reported with spermatogenesis. *RNF17* was essential for spermiogenesis (Pan et al., 2005) and *OPRL1* was exclusively expressed in the spermatocytes (Eto, 2015). *CDKN2C* gene played an important role in spermatogenesis in the adult testis (Xu et al., 2013) and *TCEA2* is expressed primarily in the testis in humans (Weaver & Kane, 1997). *SOX18* was more strongly expressed in spermatogonial cells and spermatocytes than in spermatids and testicular somatic cells of sheep (Yang et al., 2021). *ATP12A* played a role in acrosome reactions (Favia et al., 2022). *BCL2L11* affected sperm apoptosis (Ding et al., 2021). *HIBADH* was involved in the mitochondrial function of spermatozoa, and maintained sperm motility related to sperm motility (Tasi et al., 2013). *STARD9* was associated with asthenospermia (Mao et al., 2011). *PSPC1* regulated nuclear events during spermatogenesis (Myojin et al., 2004). *NPBWR2* played an essential role in hormone secretion in pigs (Yang et al., 2018). *DNAJB14* was related to heat shock protein in spermatozoa (Bansal et al., 2015). *MRPS26* was consistently expressed throughout early embryogenesis with little stage or tissue specificity (Cheong et al., 2020). *H2AFZ* gene had been reported as candidate gene for boar semen quality traits (Zhao et al., 2019). *UNC5C* exerted significant effects on the conception rate in Holsteins (Sugimoto et al., 2015). *ABHD16B* was involved in bovine conception rate and sperm plasma membrane lipid composition (Shan et al., 2020). *STMN3* was identified to be potentially related to egg production in chicken (Chen et al., 2021). These results were helpful to explain the genetic mechanism of hyper‐prolificacy trait in Hu sheep.

In addition to hyper prolificacy, Hu sheep have other breed‐specific traits. Hu sheep are also well recognized for the beautiful wavy lambskins, and their fleeces consist of white coarse wool and heterotypical fibers. Moreover, they possess a short fat tail and are round with the tip upward‐pointing. In this study, two genes (*LAMTOR3* and *EEF1A2*) identified were associated with hair follicle development. *LAMTOR3* gene was reported to be associated with the development of hair follicles and cashmere growth in Liaoning cashmere goats (Jin et al., 2019). *EEF1A2* gene has been demonstrated to be related to wool‐related traits in sheep by a phenome‐wide association study in fine‐wooled Merino sheep (Zhao et al., 2021). Genes *HOXA10* and *HOXA11* identified in OAR4 may play important roles in fat tail formation. Both two genes were identified by selection signature detection in Chinese indigenous sheep breeds with different types of tails (Li et al., 2020; Liu et al., 2021; Yuan et al., 2017; Zhao et al., 2020) and *HOXA10* was further validated by RNA Seq as a candidate gene strongly linked with fat deposition in sheep tails (Bakhtiarizadeh & Alamouti, 2020). *HOXA1* and *KCNQ2* have been documented as being associated with ear size in sheep (Li et al., 2020; Qiao et al., 2015).

Hu sheep can also be used for producing mutton. Some of the candidate genes have been documented as important candidate genes for development and fat metabolism as well. *FAF1* gene was associated with dysfunctions of bone and joints in pigs and can lead to osteochondrosis (Rangkasenee et al., 2013). *CCNDBP1* was considered as a new positive regulator of skeletal myogenesis (Huang et al., 2016). *GJB2* and *GJA3* had been reported to be related to body size and developments, were identified by selection signature detection in sheep (Kim et al., 2016) and also resided in the ROH islands in Chinese sheep breeds (Abied, Xu, et al., 2020; Liu et al., 2021). *ACOXL* had documented association with fat metabolism (Onteru et al., 2013). *JAZF1* was shown to regulate lipid metabolism (Ming et al., 2014) and was also reported to be related to height in humans (Johansson et al., 2009). Moreover, this gene was identified by genome‐wide selection signature detection in cattle (Zhao et al., 2015) and sheep (Zhao et al., 2020). Both *HOXA3* and *HOXA5* genes were involved in controlling the development of the subcutaneous abdominal adipose tissues (Karpe & Pinnick, 2015). *HOXA4* was considered as a transcriptional regulator of terminal brown fat cell differentiation (Pradhan et al., 2017), *EBF4* was identified to be associated with body fat traits in humans by a genome‐wide methylation study (Cao et al., 2021).

In addition, we identified other candidate genes related to immune and feed intake. *UBR1* was potentially related to trypanotolerance, since the previous study had shown that E3‐ubiquitin ligases played a decisive role in the immune response (Serranito et al., 2021). *FASTKD5* interacted with *NLRX1* to promote HIV1 replication (Guo et al., 2021). *DAPP1* has been reported to be associated with behavior and/or feed intake traits in pigs (Do et al., 2013), and *RNF17* has also been reported to be involved in feed intake in Pekin Ducks (Zhu et al., 2019). *NPBWR2* could inhibit the release of growth hormone and prolactin in chicken pituitary cells and played a significant role in the hypothalamic control of feed intake and hence energy homeostasis (Scanes, 2016).

## 5 CONCLUSION

In this study, we applyed three complementary test statistics to identify the selection signatures within Hu sheep using the whole genome sequencing data. A total of 10 significant genomic regions were simultaneously identified by multiple methods. These genomic regions harbored 92 candidate genes. Out of them, 32 genes associated with reproduction were resided in 9 genomic regions under selection. Among them, two star genes (*BMPR1B* and *GNRH2*) and one HOXA gene cluster (*HOXA1*‐*5*, *HOXA9*, *HOXA10*, *HOXA11* and *HOXA13*) have been documented to be associated with fertility. Moreover, we identified other genes that are related to hair follicle development, ear size, fat tail formation, growth and development, fat deposition, immune and feed intake. Our results could contribute to understanding the genetic mechanisms underlying the selection of breed‐specific traits in Hu sheep, and provide a reference for genetic improvement program in Hu sheep.

## FUNDING INFORMATION

This research was funded by the Natural Science Foundations of China (No. 32172702), the National Key Research and Development Program of China (2021YFD1301101), and the Agricultural Science and Technology Innovation Program (ASTIP‐IAS02).

## CONFLICT OF INTEREST STATEMENT

The authors declare no conflict of interest.

## Supporting information


Figure S1.

Table S1.

Table S2.

Table S3.

Table S4.

## Data Availability

Data available from the Dryad Digital Repository: https://DOI: 10.5061/dryad.rbnzs7hkv.
