# Supplementary figures and images for: Analysis of 206 whole‐genome resequencing reveals selection signatures associated with breed‐specific traits in Hu sheep
Source: Evol Appl. 2024 Jun 21;17(6):e13697. doi: 10.1111/eva.13697 (PMC11192971; doi:10.1111/eva.13697)

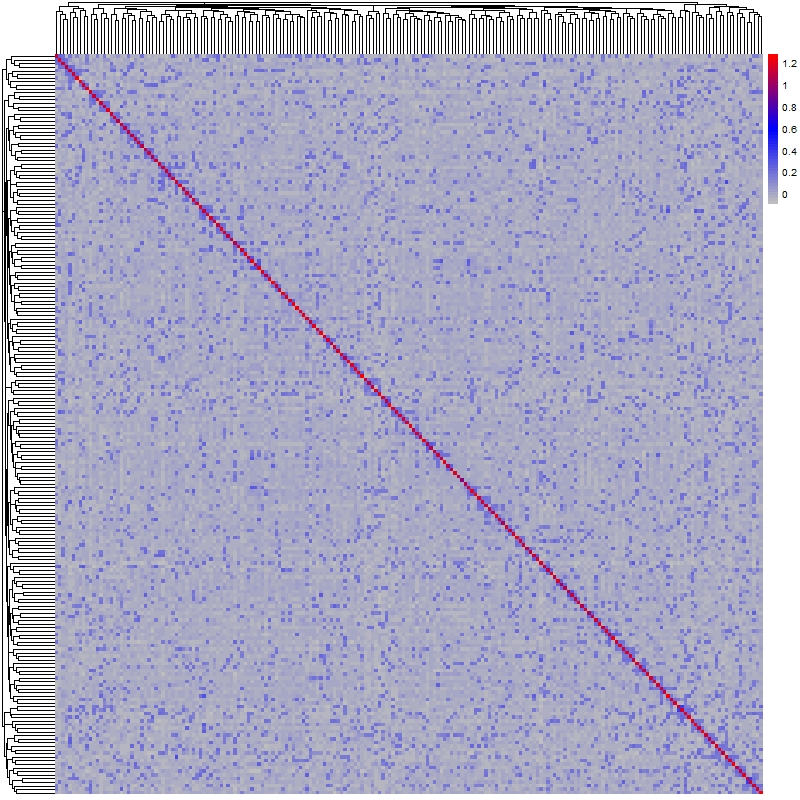


Fig S1 heatmap of genomic relationships between all sheep analyzed

Supplement: Supplementary file 1 — Figure S1. [file EVA-17-e13697-s002.docx]
